# Respiratory Transmission Potential of Chikungunya Virus: Integrating Aerosol Stability, Clinical Evidence, and Mechanistic Insights

**DOI:** 10.3390/microorganisms14071514

**Published:** 2026-07-11

**Authors:** Tao-An Chen, Sui-Loi Mak, Ya-Ting Chuang, Yu-Hsiang Hsu

**Affiliations:** 1 Division of Respiratory Therapy, Department of Chest Medicine, Show Chwan Memorial Hospital, Changhua City 500009, Changhua County, Taiwan; b117100045@tmu.edu.tw; 2 Department of Critical Care Medicine, Show Chwan Memorial Hospital, Changhua City 500009, Changhua County, Taiwan; 3 Surgical Intensive Care Unit, Department of Nursing, Show Chwan Memorial Hospital, Changhua City 500009, Changhua County, Taiwan; s09901383@cjc.edu.tw

**Keywords:** Chikungunya virus, Chikungunya fever, respiratory tract infections, respiratory system, aerosols, disease transmission, virus shedding, nonhuman primates

## Abstract

Chikungunya virus (CHIKV), traditionally recognized as a mosquito-borne alphavirus that causes febrile illness and debilitating arthralgia, has increasingly been associated with atypical organ involvement, including respiratory manifestations. These observations raise important questions regarding whether respiratory symptoms reflect severe systemic disease or signal previously underappreciated respiratory exposure routes. This review aimed to synthesize current evidence on respiratory complications of CHIKV infection and to evaluate the plausibility of respiratory or aerosol-associated transmission. A systematic literature search of PubMed, EMBASE, and MEDLINE (Ovid) identified five eligible studies spanning clinical virology, outbreak surveillance, epidemiology, and experimental aerosol models. Across human studies conducted in India, Réunion Island, Puerto Rico, and Brazil, respiratory presentations—including pneumonia, dyspnea, and respiratory failure—were uncommon but consistently associated with increased hospitalization and mortality risk. Respiratory symptoms generally arose in the context of respiratory viral coinfections, systemic inflammation, or cardiopulmonary decompensation rather than primary viral tropism for the respiratory tract. Only one non-human primate study directly evaluated aerosol exposure, demonstrating that cynomolgus macaques could be infected via inhaled CHIKV, confirming biological plausibility but showing no evidence of enhanced respiratory pathology. Importantly, no epidemiologic data support human-to-human airborne or droplet transmission. Collectively, available evidence indicates that respiratory involvement serves as a marker of disease severity rather than a transmission route. Nonetheless, rare aerosol-acquisition events in laboratory settings underscore the need for continued vigilance, strengthened surveillance, and re-evaluation of respiratory risks as climate change and viral evolution expand CHIKV’s global footprint.

## 1. Introduction

Chikungunya virus (CHIKV) is a mosquito-borne, single-stranded positive-sense RNA virus belonging to the Togaviridae family and genus Alphavirus [1]. It is responsible for acute febrile illness characterized by severe and often debilitating arthralgia [1]. The primary vectors for CHIKV are *Aedes aegypti* and *Aedes albopictus* [2]. As an arthritogenic pathogen capable of causing large-scale outbreaks across diverse geographical regions, CHIKV continues to pose a substantial global public health burden [3].

CHIKV was first isolated in 1952 from a febrile patient in Tanzania, and by the mid-1950s, infections were rapidly reported across several Asian countries [4,5,6,7]. Sporadic outbreaks persisted throughout Africa and Asia from the 1960s to the 1980s, after which evidence of virus circulation and disease activity gradually waned [8]. However, in 2004, CHIKV re-emerged on a large scale, marking the beginning of renewed global spread [9,10]. The 2005–2006 outbreak on Réunion Island remains one of the most notable CHIKV epidemics, characterized by an exceptionally high attack rate in which nearly one-third of the population was affected, accompanied by complex clinical manifestations and reported fatalities [9,11,12]. In 2007, CHIKV established local transmission in Europe, leading to locally transmitted cases in Italy and ultimately resulting in several hundred autochthonous infections [3,13]. Subsequently, in 2010, Guangdong Province in China experienced its first large-scale CHIKV outbreak, which later spread to multiple provinces across the country [14]. The virus was first identified in the Pacific region in 2011, followed by widespread outbreaks across several Pacific islands beginning in 2013 [3,15]. In the same year, CHIKV was reported for the first time in the Caribbean, from where it rapidly disseminated throughout the Americas [16,17]. Since the first recognized human case in 1952, CHIKV has caused outbreaks and established regional transmission on a global scale [4,18]. It is estimated that more than 75% of the world’s population now resides in areas at risk of CHIKV infection [18].

Currently, CHIKV is classified into four major genotypes—West African (WA), East/Central/South African (ECSA), Asian (AsU), and the Indian Ocean Lineage (IOL)—a genomic diversity that reflects the virus’s historical geographic distribution and its subsequent evolutionary expansion [19]. At the same time, global warming and rapid urbanization—combined with the widespread activity of Aedes mosquitoes and high population density—have further amplified the global risk of CHIKV transmission [19,20]. As global population density continues to rise and CHIKV transmission dynamics evolve through ongoing viral adaptation, concerns surrounding alternative or previously underrecognized transmission routes have become increasingly relevant. Advances in modern biotechnology—including high-resolution genomic surveillance, viral aerosol characterization, and improved detection of low-level respiratory shedding—now allow for a more precise assessment of these potential risks [21,22,23]. Given these developments, it is essential to re-examine whether CHIKV may possess any capacity, however limited or context-dependent, for respiratory or aerosol-associated transmission. To address this question, we conducted a systematic review synthesizing current evidence from epidemiological observations, laboratory studies, and mechanistic investigations.

## 2. Materials and Methods

### 2.1. Search Strategy

We conducted the literature search in accordance with the Preferred Reporting Items for Systematic Reviews and Meta-Analyses (PRISMA) guidelines [24,25]. A comprehensive search was performed in PubMed, EMBASE, and MEDLINE (Ovid) to identify publications relevant to Chikungunya virus (CHIKV) infection, respiratory involvement, and potential respiratory transmission pathways. The search strategy encompassed three conceptual domains: (i) Chikungunya virus infection, incorporating controlled vocabulary and free-text terms related to Chikungunya, Chikungunya virus, and CHIKV; (ii) respiratory manifestations, including terminology describing respiratory symptoms, respiratory tract infections, and lower respiratory complications such as pneumonia, dyspnea, and cough; and (iii) transmission risk, capturing terms associated with infectious disease spread, including aerosol and droplet transmission, airborne mechanisms, and human-to-human transmission. The search was limited to English-language publications available up to 31 October 2025. The Systematic Review has been registered in the Open Science Framework (https://osf.io/4w9x5; accessed on 11 July 2026).The completed PRISMA 2020 checklist is provided in the Supplementary Materials (Supplementary File S1).

### 2.2. Eligibility Criteria

We included peer-reviewed studies that examined clinical, epidemiological, or experimental evidence related to respiratory manifestations or potential respiratory transmission pathways of CHIKV. Eligible studies met the following criteria: (i) investigations involving human participants of any age with confirmed or suspected CHIKV infection, reporting respiratory symptoms, respiratory complications, atypical or severe disease presentations, or clinical outcomes such as hospitalization, intensive care admission, or mortality; (ii) epidemiological studies analyzing risk factors for severe or fatal CHIKV infection, with particular attention to respiratory system involvement; (iii) experimental or preclinical studies assessing respiratory tract infection, aerosol exposure, or respiratory pathophysiology in CHIKV-infected animal models; and (iv) studies providing extractable data on respiratory features, clinical severity, transmission routes, or host factors associated with atypical or severe CHIKV infection. We excluded review articles, case reports, conference abstracts, editorials, commentaries, and studies lacking primary data on respiratory involvement or disease severity.

### 2.3. Study Selection and Data Extraction

Two reviewers (T.-A.C. and S.-L.M.) independently screened the titles and abstracts of all retrieved records after the database search. Articles deemed potentially eligible were retrieved for full-text review. Any disagreements during the selection process were resolved through discussion, and a third reviewer (Y.-H.H.) was consulted when necessary. When essential information was missing or unclear, attempts were made to contact the original study authors.

## 3. Results

### 3.1. Characteristics of Included Studies

**Figure 1:**
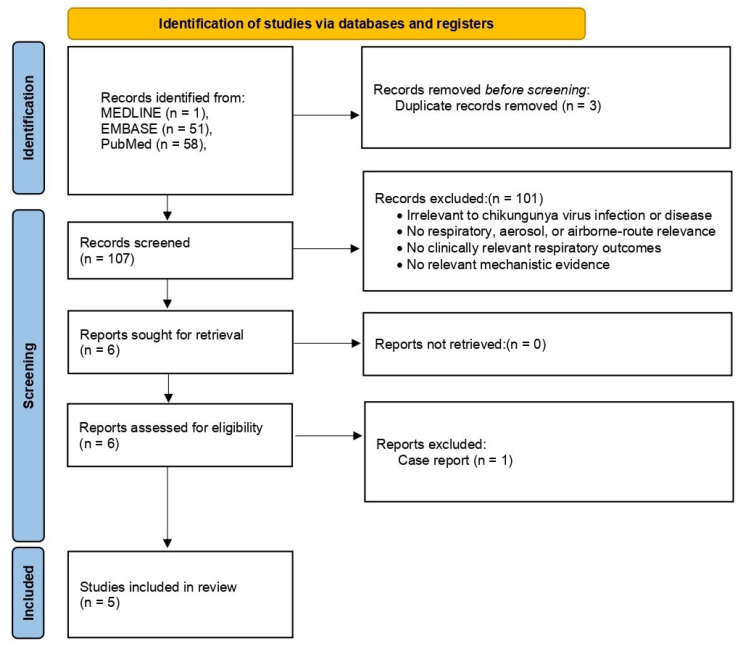
PRISMA 2020 flow diagram of study identification, screening, eligibility assessment, and inclusion.

The schematic diagram of the study screening process is presented in Figure 1. A total of five studies met the inclusion criteria for this review, representing diverse methodological approaches and spanning multiple geographic regions (Table 1). The included research was conducted across India (Pondicherry/Karaikal), Réunion Island (a French overseas region in the Indian Ocean), the United States (continental U.S. and Puerto Rico), and Brazil (Fortaleza) [12,23,26,27,28]. The methodological designs varied considerably, comprising one cross-sectional clinical virology investigation, one hospital-based surveillance cohort, one non-human primate (NHP) experimental aerosol-challenge study, one retrospective sentinel-surveillance analysis, and one matched case–control study assessing fatal outcomes [12,23,26,27,28]. Collectively, these studies examined respiratory manifestations, atypical clinical presentations, and potential transmission-related characteristics of CHIKV in both human and non-human models [12,23,26,27,28].

Four studies were conducted in human populations across Asia, Europe, and the Americas [12,26,27,28]. A cross-sectional virology study from India evaluated 69 cases out of 110 suspected individuals presenting with CHIKV-compatible symptoms [26]. In Réunion Island, a hospital-based surveillance study analyzed 610 adult patients with atypical presentations during the 2005–2006 outbreak, providing one of the earliest large-scale descriptions of severe or organ-specific involvement [12]. A retrospective sentinel surveillance study in Puerto Rico assessed 1469 PCR-confirmed CHIKV infections and contributed evidence on clinical manifestations during the 2014 epidemic [27]. One study specifically examined fatal outcomes. A matched case–control study from Fortaleza, Brazil, compared 82 fatal CHIKV cases with 164 survivors, offering valuable insights into risk factors for severe disease and potential complications [28].

In addition to human data, one non-human primate (NHP) experimental challenge model was included which exposed 12 cynomolgus macaques to CHIKV via aerosol and intradermal routes, providing mechanistic insights relevant to potential respiratory exposure pathways and host–pathogen interactions [23].

### 3.2. Respiratory Manifestations Across Human CHIKV Studies

Respiratory involvement was documented across several human studies, though its clinical significance varied by setting and population characteristics [12,26,27,28]. A cross-sectional virology study reported that respiratory symptoms in CHIKV cases were predominantly attributable to RSV co-infection (87%), with only 4% representing cases without identified co-infection—highlighting the importance of respiratory viral co-pathogens during CHIKV outbreaks [26]. In contrast, during the 2005–2006 Réunion Island epidemic, a study identified substantial pulmonary disease within a large cohort of 610 atypical adult cases, including 17% with pneumonia and 8% with respiratory failure, both strongly associated with severe disease and mortality [12]. Similar patterns emerged in the Puerto Rico sentinel surveillance data, where among 1469 PCR-confirmed cases, respiratory signs independently increased the risk of hospitalization [27]. Moreover, the Brazil case–control study demonstrated that dyspnea was one of the strongest predictors of death (OR 50.61), emphasizing that respiratory distress functions as a key indicator of systemic severity rather than primary respiratory tropism [28]. These findings from India, Réunion Island, Puerto Rico, and Brazil indicate that respiratory symptoms in CHIKV infections arise mainly in the context of co-infections, systemic inflammation, or decompensation of underlying cardiopulmonary conditions—yet consistently correlate with worse clinical outcomes [12,26,27,28].

### 3.3. Evidence Evaluating Respiratory Transmission of CHIKV

Only one study directly investigated the potential for respiratory transmission [23]. The experimental aerosol challenge exposed cynomolgus macaques (n = 12) to aerosolized CHIKV and demonstrated successful viral infection via the respiratory route, establishing biological plausibility for aerosol acquisition [23]. However, respiratory illness in exposed primates remained mild or absent, and the clinical and virological course resembled that seen after intradermal infection [23]. Crucially, no human studies to date have corroborated airborne, droplet, or other respiratory-mediated spread of CHIKV, including during high-intensity outbreaks with comprehensive surveillance (e.g., Réunion Island) [12,27]. Evidence therefore supports experimental susceptibility without translation to observed human-to-human respiratory transmission [12,23,27].

### 3.4. Integrated Interpretation of Respiratory Risks and Transmission Potential

Taken collectively, current evidence suggests that respiratory manifestations represent a clinical severity signal rather than a transmission mechanism [12,26,27,28]. Across multiple outbreaks, dyspnea, respiratory failure, and pneumonia consistently predicted hospitalization and mortality, yet these symptoms did not indicate respiratory spread [12,26,27,28]. The only confirmed respiratory-route infection model remains non-human primate aerosol exposure, which supports theoretical feasibility but lacks epidemiologic confirmation [23]. Overall, respiratory involvement in CHIKV infection should be interpreted primarily as an indicator of co-infections, systemic inflammation, cardiopulmonary decompensation, or severe atypical disease, rather than evidence of respiratory transmission. Current data continue to support vector-borne transmission as the exclusive driver of human CHIKV spread.

## 4. Discussion

Climate warming has expanded environmentally suitable habitats for Aedes mosquitoes, facilitating their migration into higher-latitude regions [29]. Moreover, climate change—when combined with rapid urbanization and increasing population density—has further amplified the regional risk of CHIKV transmission [19]. Clinical presentation of Chikungunya virus-induced disease (CHIKVD) typically includes fever and musculoskeletal pain [3,12]. Following an incubation period of approximately 1–12 days, most patients develop acute symptoms such as high-grade fever (often >39 °C/102 °F), arthralgia, headache, fatigue, and myalgia, all of which may be profoundly debilitating [3,12,28]. Arthralgia represents the hallmark of CHIKVD and is characteristically bilateral and symmetric, affecting corresponding joints on both sides of the body—including those of the hands, feet, knees, and wrists [3,12,28]. This joint involvement is attributed to the virus’s tropism for synovial tissues, which triggers immune-mediated inflammation and severe pain [3,30]. In more complex or severe cases, CHIKV can also produce neurological and cardiovascular manifestations [12,28,31]. Increasing reports from clinical case studies further suggest that respiratory symptoms and potential transmission-related concerns are emerging areas of relevance [23,26,27,28].

CHIKV exhibits substantial genetic diversity and is currently classified into four major lineages: West African (WA), East/Central/South African (ECSA), Asian (AsU), and the Indian Ocean Lineage (IOL) [19,32,33]. These lineages differ not only in their nucleotide and amino acid compositions but also in the length and structural organization of their 3′ untranslated regions (3′UTRs) [32,33]. Variations within the 3′UTR play a critical role in regulating viral replication and adaptation within Aedes mosquito vectors [32,33]. Over the past several decades, CHIKV has undergone extensive evolutionary diversification, resulting in the emergence of multiple viral strains with enhanced transmissibility and virulence [3,19,33]. The most well-recognized adaptive mutation is the single-point substitution in the E1 glycoprotein (A226V), which emerged during the 2005–2006 outbreak on La Réunion Island in the Indian Ocean and substantially enhanced the virus’s fitness in Aedes albopictus mosquitoes [12,34]. In addition, naturally occurring and experimentally observed mutations in CHIKV continue to evolve, further increasing the virus’s adaptability and pathogenic potential [35,36]. Taken together, as climate change and urban expansion continue to accelerate, these forces—coupled with increasingly efficient vector-mediated transmission—are driving a sustained rise in CHIKV activity.

Globally, as the incidence of CHIKV infection continues to rise, greater attention must be paid to its atypical clinical presentations [12,27,28]. Atypical cases can broadly be categorized into three groups: (i) exacerbation of pre-existing medical conditions, (ii) unmasking or worsening of previously unrecognized disorders, and (iii) exaggerated or organ-specific manifestations directly attributable to viral infection [12]. During the 2005 outbreak on La Réunion Island, the incidence of atypical CHIKV infection was approximately 0.3%, yet nearly 40% of these patients required admission to the intensive care unit (ICU) [12,37]. Although atypical presentations accounted for only a small proportion of total cases, their mortality rate was unexpectedly high, marking the first documented fatalities associated with CHIKV infection [26,38]. Notably, it was during this outbreak that pneumonia and other lower respiratory tract manifestations were first recognized by clinicians as part of the expanding clinical spectrum of CHIKVD [12]. At the same time, other surveillance data have demonstrated that clinically significant pulmonary involvement—including pneumonia and respiratory failure—can occur in atypical or severe presentations, highlighting that CHIKV may contribute to lower respiratory tract complications under specific physiological or immunological conditions [12,23,26,27,28]. Additional epidemiologic analyses further indicate that respiratory signs independently predict increased likelihood of hospitalization, while respiratory distress has emerged as one of the strongest clinical markers associated with fatal outcomes [27,28].

Emerging evidence suggests that severe pulmonary involvement—including acute respiratory distress syndrome (ARDS)—can occur, particularly during large outbreaks or in patients with underlying comorbidities [39]. Although case reports and case series were excluded from the systematic evidence synthesis, they are discussed here as supplementary contextual evidence to illustrate rare but clinically important respiratory complications reported in CHIKV infection. Adult case reports have described abrupt hypoxemia, bilateral infiltrates, and PaO_2_/FiO_2_ ratios consistent with ARDS after exclusion of alternative infectious etiologies, with imaging frequently demonstrating diffuse ground-glass opacities or alveolar hemorrhage [39,40,41]. Some patients improved only after corticosteroid therapy, underscoring an inflammatory component of lung injury [41]. Pediatric cases, though rare, reinforce the possibility of severe respiratory disease across age groups [39,42]. Among 58 CHIKV-infected children, one fatal ARDS case was reported, and an infant presented with pneumonia and septic shock, indicating that respiratory failure may develop as part of systemic inflammatory involvement in vulnerable hosts [42]. Overall, CHIKV-associated ARDS is uncommon but clinically significant, with presentations ranging from viral pneumonitis to alveolar hemorrhage and multi-organ dysfunction requiring mechanical ventilation [39,40,41,42]. In the absence of standardized management guidelines, early recognition, exclusion of mimicking pathogens, and prompt respiratory support remain essential to reducing morbidity and mortality.

Taken together, these findings suggest that although CHIKV is not primarily a respiratory pathogen, respiratory manifestations—whether arising from co-infection, systemic inflammatory responses, or atypical disease trajectories—serve as important indicators of clinical deterioration [12,26,27,28]. Importantly, emerging evidence also highlights the need for continued vigilance regarding the potential for aerosol-mediated acquisition in rare or non-classical contexts [22,23,43,44]. Experimental studies and reports of laboratory-acquired infections have demonstrated that CHIKV can establish infection following inhalation of aerosolized viral particles, making it the only recognized arthritogenic alphavirus capable of transmission through this route [22,23,43]. Although documented cases remain infrequent and typically exhibit milder and shorter clinical courses compared with conventional mosquito-borne infections, the occurrence of detectable viremia after presumed aerosol exposure reinforces the biological plausibility of this pathway [23,43,44]. Therefore, in scenarios involving atypical presentations, laboratory exposures, or other high-risk environments, the possibility of aerosol transmission—albeit rare—should be considered when evaluating CHIKV-related respiratory involvement and determining the appropriate biosafety measures.

**Figure 2:**
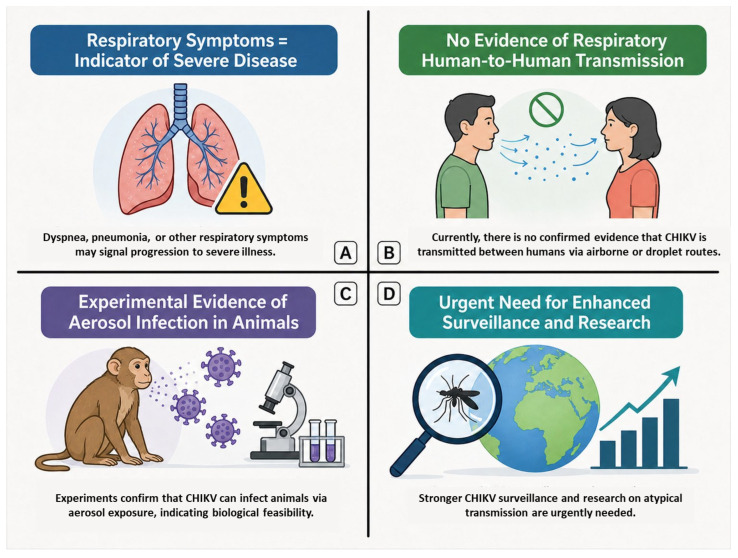
Key Insights on Respiratory Transmission Risk of CHIKV. This infographic summarizes key insights into the respiratory transmission risk of CHIKV. (**A**): Respiratory symptoms such as dyspnea and pneumonia are indicators of severe disease and are associated with higher risks of hospitalization and mortality. (**B**): There is currently no epidemiologic evidence supporting human-to-human respiratory transmission. (**C**): Experimental animal data demonstrate that aerosol infection is biologically feasible. (**D**): These findings highlight the urgent need for strengthened surveillance and further research into atypical or non-vector transmission routes.

These considerations indicate that CHIKV will remain a significant global public health threat in the coming years [19,28,29]. Climate change is irreversibly expanding the geographic range of Aedes vectors, thereby increasing the likelihood of CHIKV introduction into previously unaffected regions [45,46]. Strengthened global efforts are needed to investigate, monitor, and model the effects of climate change on vector distribution. At the same time, continued surveillance is essential to detect the emergence of genetically divergent CHIKV variants that may alter clinical presentation or potentially heighten respiratory-related risks [27,28,45,46]. Based on the above discussion, we present a summary in Figure 2.

## 5. Limitations and Future Research

Several limitations should be considered when interpreting the findings of this review. First, the available evidence remains limited, with only five eligible studies identified across clinical, epidemiological, and experimental domains [12,23,26,27,28]. Second, only one non-human primate study directly evaluated aerosol exposure, and its findings may not be directly generalizable to human transmission dynamics [23]. Third, the human evidence was largely derived from heterogeneous outbreak surveillance, retrospective analyses, and observational studies, limiting causal interpretation regarding respiratory involvement [12,26,27,28]. Fourth, respiratory manifestations in CHIKV infection may be confounded by respiratory viral co-infections, systemic inflammation, underlying cardiopulmonary disease, or severe atypical disease presentations [12,26,27,28]. Fifth, the heterogeneity of study designs limited the application of a single standardized risk-of-bias tool and may have constrained the methodological rigor of the evidence synthesis. Finally, no epidemiological study has confirmed human-to-human airborne or droplet transmission of CHIKV; therefore, conclusions regarding respiratory transmission risk should be interpreted cautiously.

Future studies should adopt prospective, multicenter cohort designs to clarify the incidence, clinical spectrum, and risk factors of respiratory manifestations in CHIKV infection. Standardized case definitions, systematic respiratory pathogen testing, and clear etiological attribution are needed to distinguish CHIKV-related respiratory involvement from co-infections, hospital-acquired infections, systemic inflammatory responses, and exacerbations of underlying cardiopulmonary diseases. Long-term follow-up studies are also warranted to determine whether CHIKV infection is associated with chronic respiratory sequelae, such as persistent pulmonary function impairment, airway remodeling, bronchiolitis, or pulmonary fibrosis. In addition, further experimental and translational studies are needed to evaluate aerosol stability, respiratory tissue tropism, and the plausibility of airborne or droplet transmission under real-world exposure conditions. Because the current systematic search identified only one eligible non-human primate study directly addressing CHIKV aerosol exposure, future comparative animal studies are needed to further clarify CHIKV respiratory tropism, aerosol infection potential, and host immune responses.

## 6. Conclusions

This review highlights that respiratory involvement in CHIKV infection—although uncommon—remains clinically meaningful and warrants heightened vigilance. Human studies consistently show that dyspnea, pneumonia, and respiratory failure are strong indicators of clinical deterioration, often associated with co-infections or systemic inflammation. These manifestations underscore the need for careful respiratory assessment, particularly during large outbreaks or in vulnerable populations. Experimental data further demonstrate that CHIKV can establish infection following aerosol exposure in non-human primates, providing biological plausibility for respiratory acquisition. While no human-to-human respiratory transmission has been confirmed to date, the presence of this mechanistic pathway indicates that respiratory transmission risks—however rare—should not be dismissed, especially in laboratory, high-risk, or atypical exposure settings.

As climate-driven expansion of Aedes vectors and ongoing viral evolution continue, strengthened surveillance, rigorous respiratory monitoring, and multidisciplinary research are essential to detect early signals of changing clinical patterns and to reassess potential respiratory transmission risks over time.

## Figures and Tables

**Table 1:** Characteristics of Included Studies Examining Respiratory Manifestations or Transmission-Related Findings in Chikungunya Virus Infection.

First Author (Year)	Country	Study Design/Model	Human/ Non-Human	Sample/ Population	Respiratory Findings	Respiratory Transmission Evidence
Sankari et al., (2008) [26]	India (Pondicherry and Karaikal)	Cross-sectional virology study	Human	69 CHIKV cases (From 110 suspected CHIKV cases)	Respiratory symptoms largely due to RSV co-infection (87%), only 4% CHIKV alone	No
Economopoulou et al., (2009) [12]	France (Réunion Island)	Hospital-based surveillance study	Human	610 atypical CHIKV adult cases	Pneumonia 17%, respiratory failure 8%	No
Cirimotich et al., (2017) [23]	United States	Experimental NHP challenge (aerosol and intradermal)	Non-human	12 cynomolgus macaques	Mild or absent respiratory illness after aerosol infection	Yes; aerosol infection biologically possible
Hsu et al., (2019) [27]	United States (Puerto Rico)	Retrospective cross-sectional sentinel surveillance study	Human	1469 PCR-confirmed CHIKV cases	Respiratory findings increase hospitalization risk	No
Oliveira et al., (2022) [28]	Brazil (Fortaleza)	Matched case–control study	Human	82 fatal CHIKV cases/ 164 survival CHIKV cases	Dyspnea strongly predicted mortality (OR 50.61)	No

CHIKV: Chikungunya virus; NHP: non-human primate; OR: odds ratio; PCR: polymerase chain reaction; RSV: respiratory syncytial virus.

## Data Availability

No new data were created or analyzed in this study. Data sharing is not applicable to this article.

## Supplementary Materials

The following supporting information can be downloaded at: https://www.mdpi.com/article/10.3390/microorganisms14071514/s1. File S1: PRISMA 2020 Checklist.

## Abbreviations

The following abbreviations are used in this manuscript:

ARDS	acute respiratory distress syndrome
CHIKV	Chikungunya virus
CHIKVD	Chikungunya virus-induced disease
NHP	non-human primates
OR	odds ratio
PCR	polymerase chain reaction
PRISMA	Preferred Reporting Items for Systematic Reviews and Meta-Analyses
RSV	respiratory syncytial virus
